# Well Done, Pennsylvania!

**Published:** 1901-05

**Authors:** 


### WELL DONE, PENNSYLVANIA!

THE signal defeat in the House of Representatives of the measure destined to reopen registration of non-graduates in the Keystone State is a victory of no small moment, and the profession of Pennsylvania have earned a decisive victory in its defeat. The strong influences back of this measure, its successful passage in the Senate, its favorable report from the House Committee, were all elements of strength that made its passage more than probable. A delegation of nearly fifty members of the profession were before the House Committee, and more than five hundred active veterinarians in every section of the State placed themselves in antagonism to the measure, and their unanimous appeal for its defeat proved successful in the House. We congratulate the profession of the State in such a display of activity, and their showing of interest in all such matters for the welfare of the Commonwealth betokens advancement and progress all along the line, and maintains a high place for Keystone veterinarians among their colleagues the world over.

